# Working With People With Experience of Psychosis to Co‐Design an Educational and Anti‐Stigma Psychosis Intervention for Schools

**DOI:** 10.1111/hex.70333

**Published:** 2025-10-17

**Authors:** William John Parrott, Mike Jackson, Anne Krayer

**Affiliations:** ^1^ School of Medical and Health Sciences Bangor University Bangor UK; ^2^ North Wales Clinical Psychology programme Bangor University Bangor UK

**Keywords:** adolescent mental health, co‐design, lived experience perspective, mental health literacy, psychosis stigma

## Abstract

**Introduction:**

Psychosis is a particularly stigmatised condition in adolescent populations, with prevalent stigmatising beliefs surrounding it including negative stereotypes around dangerousness, unpredictability and chronicity. Additionally, the first episodes of psychosis increase significantly during late adolescence, and there is a lack of understanding and recognition of psychosis in young people. Given these factors, psychosis appears to be an important topic for mental health literacy and anti‐stigma programmes. However, there is a lack of consensus surrounding the best way to construct psychosis anti‐stigma interventions for young people. Voices of people with experience of psychosis seem crucial in this regard, but their experiences of stigma and discrimination are rarely heard. By including their perspectives along with those of other stakeholders, we aim to ensure that we gain a better, more balanced perspective on how to talk about psychosis with young people.

**Methods:**

This study reports on the co‐design process of a psychosis anti‐stigma educational intervention for young people using the short‐form Guidance for Reporting Involvement of Patients and the Public (GRIPP‐2) checklist as a guiding framework. Six workshops were held over 3 months, with experts with experience of psychosis working with clinicians and academics. These workshops explored the differing views of these groups on how we understand, talk about and perceive psychosis.

**Results:**

This process resulted in an educational module named ‘Reality and Psychosis’, which we believe will prove effective in not only educating but also diminishing stigma surrounding psychosis among young people.

**Conclusion:**

By incorporating various perspectives, we hope to provide a well‐rounded and balanced approach to addressing the complexities of discussing psychosis with young people. The GRIPP‐2 checklist also proved to be a useful framework for assisting the reporting process.

**Patient or Public Contribution:**

Individuals with lived experience of psychosis participated throughout the co‐design process. Specifically, they contributed to the six workshops by sharing their personal experiences of stigma, discrimination and recovery, which informed the development of the educational module. This helped ensure the voices of those directly affected by psychosis were authentically represented.

## 1 Introduction

### 1.1 Psychosis

Psychosis is a particularly stigmatised condition in both adult and adolescent populations, with prevalent stigmatising beliefs surrounding it, including stereotypes around dangerousness, unpredictability and chronicity [1, 2, 3, 4]. Mental illness stigma significantly contributes to social exclusion and distress [5, 6, 7] and can result in less engagement with mental health services [8], internalised stigma, decreased self‐worth and self‐efficacy, and impediment of recovery [9]. It is particularly troubling that attitudes to psychosis appear to have worsened in the past 30 years, compared to other mental health conditions, which have become less stigmatised or remained at the same level of stigma [10, 11]. As the incidence of first episodes of psychosis increases significantly during late adolescence [12, 13], and understanding and recognition of psychosis is generally poor in young people [14, 15, 16], psychosis appears to be an important topic for mental health literacy and anti‐stigma programmes. However, results from systematic reviews on psychosis anti‐stigma interventions in schools [17, 18] indicate that there is little consensus on what the important components of mental health anti‐stigma interventions are and how best to construct these interventions.

### 1.2 Messaging Around Psychosis

Furthermore, attention needs to be paid to what messaging accompanies psychosis educational anti‐stigma programmes, as some interventions have been shown to increase social distance and reinforce certain stereotypes [19, 20]. For example, biogenetic messaging around mental disorders, framing psychosis as resulting from dysregulation of neurotransmitters and structural brain abnormalities, has been shown to be largely ineffective in reducing stigmatising attitudes. This approach may, in fact, increase the notion of ‘otherness’ and encourage stereotypes around poor prognosis, unpredictability and dangerousness, leading to increased desire for social distance [21, 22, 23]. This is of particular concern, as many anti‐stigma initiatives explain psychosis in these terms [23, 24, 25]. However, there are several different approaches to framing mental illness and psychosis [26]. Psychosocial messaging has been shown to be more effective in reducing stigma in areas where biogenetic explanations often fail [23, 27]. Additionally, continuum messaging around psychosis, presenting psychotic symptoms as existing along a spectrum in the general population, as opposed to a discrete ‘all‐or‐nothing’ category, may also have a role in reducing stigma [28]. As with intervention components, there is a diverging opinion on how to frame psychosis interventions, and primary research would be beneficial in elucidating this.

#### 1.2.1 The Role of Co‐Production

Engaging and involving patients and the public in the development and design of research, public services and programmes is becoming increasingly commonplace and seen as a standard of good practice [29, 30, 31]. Service users have unique insights and knowledge about the issues [32] and their involvement has been shown to lead to more relevant research questions, more user‐friendly information, better implementation of research findings and improved recruitment [33, 34]. By including their perspectives and voices alongside those of other stakeholders, such as people working in the mental healthcare system (including funders, providers, clinicians and researchers) and young people themselves, we can ensure that we gain a better, more balanced perspective on how to talk about psychosis with young people.

Moving beyond consultation, current research emphasises deeper and more meaningful participation through ‘co‐approaches’ [35]. These approaches share core principles: power‐sharing in decision‐making, valuing diverse perspectives, reciprocal benefit and equitable relationships [36]. Co‐approaches fundamentally address power imbalances by increasing public/patient involvement in decision‐making [37]. PPIE (Patient and Public Involvement and Engagement) varies in its extent, potentially spanning the entire process from commissioning to outcome assessment [38]. While terminology can overlap, approaches are broadly categorised by participation level into co‐production (full collaboration), co‐creation (knowledge generation) and co‐design (intervention development) (Grindell et al., 2002). Several examples illustrate the application of co‐approaches with individuals who have experienced psychosis [39, 40, 41].

#### 1.2.2 Purpose and Scope of This Paper

This paper reflects on the co‐design process and challenges of creating an anti‐stigma module for young people in secondary schools, guided by a framework proposed by Blomkamp [42]. Schools have been recognised as suitable environments for diminishing mental illness stigma, preventing mental health issues and promoting positive mental health practices [43]. Young people dedicate a significant portion of their lives to school activities and are accustomed to receiving information in this environment. Schools can play a crucial role in reducing mental health risk and building resilience, with many mental health initiatives in schools demonstrating impactful change [44]. A number of reviews have informed our co‐design process. Firstly, a systematic review by the authors [18] explored commonalities in messaging and components of psychosis anti‐stigma intervention in schools. Findings indicated the importance of a recovery‐oriented approach using psychosocial explanations of psychosis and that educators should be honest and open when portraying psychosis. Additionally, a wide variety of components were present in school‐based psychosis anti‐stigma interventions, which included but were not limited to: challenging myths and negative stereotypes [45, 46, 47, 48, 49], symptoms and treatment [20, 46, 48, 50], and personal experience of stigma and discrimination [20, 45, 46, 48]. Other topics covered to a lesser extent were age of onset, prevalence and the role of stress. A second systematic review was carried out exploring predictors of mental illness stigma in adolescents [51] to explore ways in which module effectiveness could be altered by individual differences. Results showed that positive peer norms were linked to lower mental illness stigma, while categorical beliefs increased stigma compared to continuum beliefs, which reduced it. Empathy and emotional distress had inconclusive effects.

**Figure 1:**
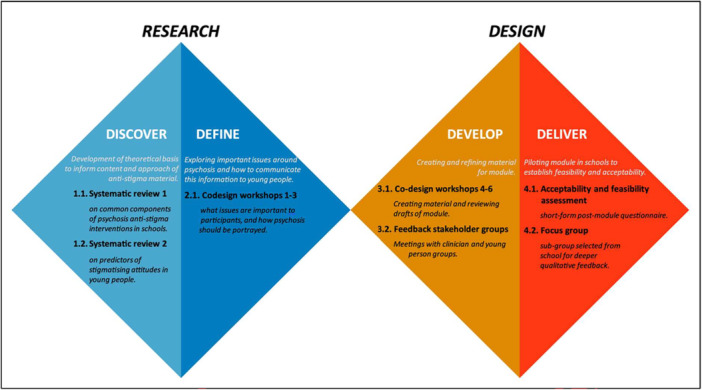
The design process illustrated using the ‘Double Diamond’ model [42].

Feedback from local and national Early Intervention groups, as well as other clinicians and academics, was sought after the co‐design process. This study reports on the development of the module, using a reflective narrative of the workshop processes, alongside an overview of the outputs produced—written first‐hand accounts of the experience of the workshops, which were then circulated and discussed within the research team and workshop participants. At this stage, participants and members of the research team were able to respond to what was written. Although working from a co‐approach perspective, there were barriers to full participation in terms of power sharing and decision‐making. This was partially due to resource constraints, but also because the co‐design stage only made up a portion of a larger project (initial research and evaluation). The research reported here is part of a PhD project, which in itself was not co‐produced. Thus, the process we used is best classified as a ‘co‐design’ approach [52]. The design of the overall project is shown in Figure 1.

### 1.3 Method

#### 1.3.1 Design

**Figure 2:**
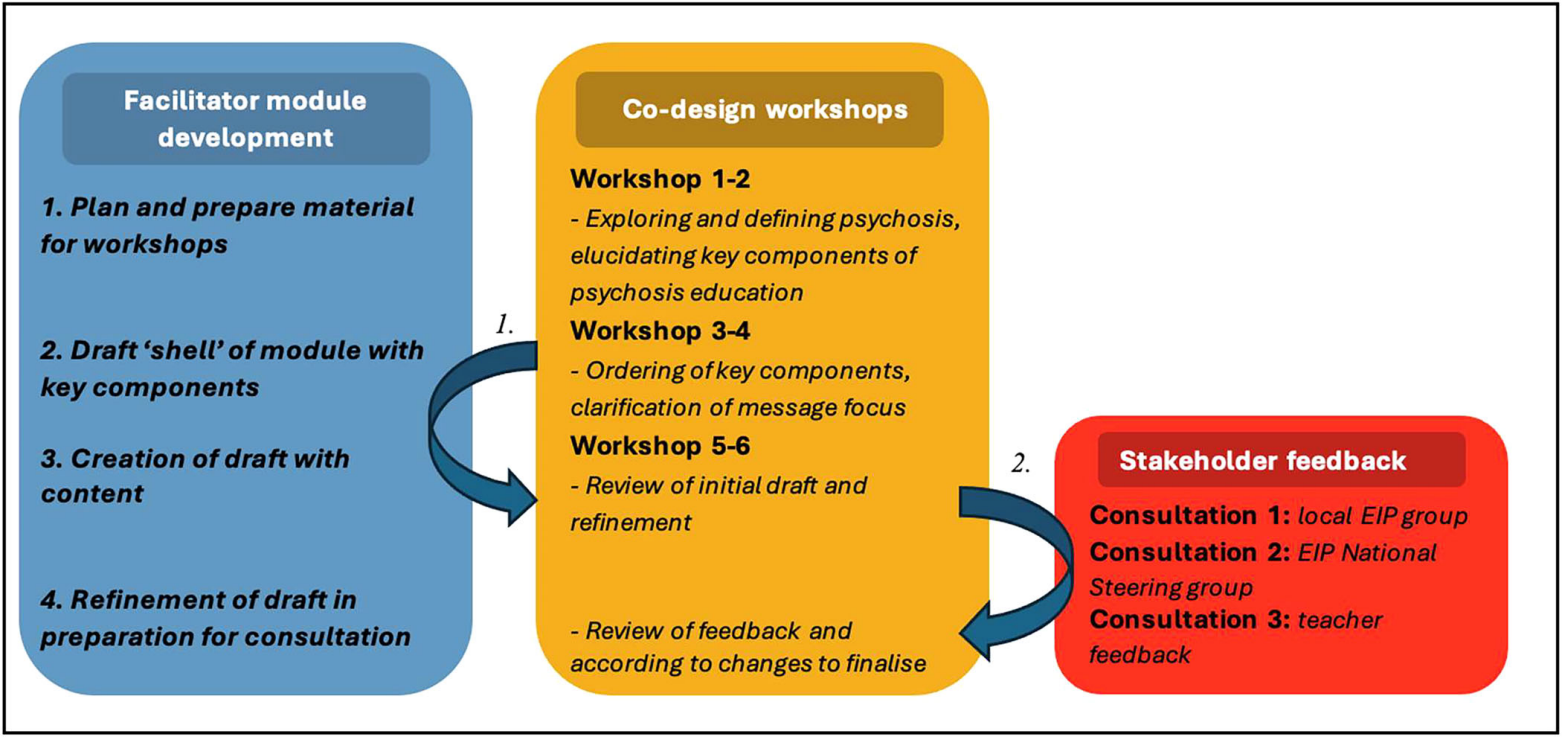
The module building process. n.b. arrows indicate (1) work done by facilitator—outside of workshops to outline and draft modules, from Workshops 3 to 6, and (2) consultation with stakeholders, with feedback then presented back to workshop participants.

The co‐design process was guided by the UK Design Council's ‘Double Diamond’ model [42]. This method was chosen as it allowed for a clear, flexible and iterative approach to creating our module. The Double Diamond model was specifically designed to facilitate exploration of larger themes in a given topic (known as divergent thought in the model) and subsequent distilling of these themes as output in the subsequent stages (convergent thought) [53]. The process has previously been used to guide the co‐design of various health interventions [54, 55, 56]. Several toolkits and guidance documents were examined and utilised to assist planning for the initial workshops [57, 58, 59]. Additionally, a review on co‐producing research with people with experience of psychosis [60] was useful in informing the design of the workshops, ensuring that key features of co‐production were captured and that potential barriers to participation were identified. The initial stages of the workshop were planned, with the co‐design process following the stages outlines in Figure 2, which were: (1) Exploring and defining psychosis, and elucidating key components of psychosis education; (2) ordering of components and message focus; (3) creation of initial draft; (4) review of initial draft and refinement; and (5) consultation with stakeholder groups.

The Guidance for Reporting Involvement of Patients and the Public (GRIPP‐2) checklist [61] was used to guide the reporting of this process. The GRIPP‐2 was developed to standardise reporting on and enable clarification of PPIE in research. The GRIPP‐2 short form was judged as suitable for use, as PPIE was not the main focus of the study, as per the checklist authors' suggestion. The short checklist looks for a reporting of the aim of PPIE involvement, the methods used to carry it out, the outcome of PPI in the study (*results*), discussion on how PPI influenced the study, and critical reflections on the process (*Experiences of PPIE*). The final three stages must include both positive and negative outcomes to provide a realistic picture of the process. GRIPP‐2 has been used to report co‐approach processes in numerous participatory research studies [62], as well as specifically with mental health experts‐by‐experience groups [63].

#### 1.3.2 Co‐Design Workshops With Experts by Experience

The workshops and subsequent consultations were approved by Bangor University College of Arts, Humanities and Business (CAHB) Research Ethics Committee (REC reference number: 2022‐17159) and Health Research Authority (HRA IRAS project ID: 315092). The workshops were facilitated by M.J., a consultant clinical psychologist, with the support of a trainee clinical psychologist and a PhD student (W.P.). Inclusion criteria for participants were that they were over the age of 18, were in contact with local (North Wales) EIP services, and were assessed to have the capacity to consent. This assessment involved discussion between the potential participant and their contact in the local services about whether they would like to participate, and if they did, whether they felt they had the emotional and mental capacity to do so. Participant well‐being was monitored throughout the process by co‐design facilitators. Participants were compensated for their attendance and were reimbursed for their travel expenses according to university guidelines. The co‐design process was directed by a group comprising these participants, working alongside the facilitators.

To maximise engagement, workshops were held at irregular intervals at the convenience of most participants, and six workshops were held over a period of 3 months. At each workshop, participants were given a choice of whether to have the subsequent workshop face‐to‐face or online, with the final workshop being facilitated online. Additionally, several workshops were delivered in premises nearer to the participants for ease of access. Due to scheduling conflicts, the number of participants varied (*n* = 2–5) in each workshop. Although there was an initial guide plan for each workshop, along with some pre‐prepared activities, these were merely suggestions, and input was sought from participants to set the agenda for each workshop.

At the end of the first three workshops, the key ideas and talking points were summarised by a notetaker. This allowed participants to contribute to this summary, to ensure it more accurately reflects the consensus of the group. W.P. updated materials based on discussions after each workshop and shared revised materials in the next workshop. This ensured that the module creation progressed in a timely manner and that the content represented participants' evolving understanding of the topic. This process allowed for an iterative design of the module, both in terms of content and design.

### 1.4 Consultation With Other Groups

After the six workshops were complete, consultation was sought from various groups, including a group of local Early Intervention clinicians, members of the Early Intervention Wales National Steering Group, and a local PSE teacher. Apart from the local Early Intervention team assisting with recruitment for the workshops, these groups had no prior involvement with the project. A draft of the module was presented to the groups, and feedback was sought on the acceptability of content, language, design and general cogency of the material. This process helped to identify any issues (and solutions) before the piloting of materials in schools. An additional review session was arranged with the original workshop group to discuss the feedback and suggested changes.

### 1.5 Analysis of Workshop

Field notes were taken during all workshops, and summaries of the main talking points were transcribed. Reflective accounts of the emerging processes and dynamics of the workshops, and the outputs created during these workshops, were written up by—and reviewed by the other researchers along with the experts‐by‐experience. A summary of these accounts appears in the results below in chronological order. Information on initial feedback from several stakeholder groups is also included. The workshops have been divided into three sections representing the different stages of the co‐design process and are accompanied by reflections by the researchers.

## 2 Results

### 2.1 We Set Out the Co‐Design Process of the Content First, Before Reflecting on the PPIE Element of the Study

#### 2.1.1 Workshops 1–2: Exploring Experiences and Challenges of Psychosis

**Figure 3:**
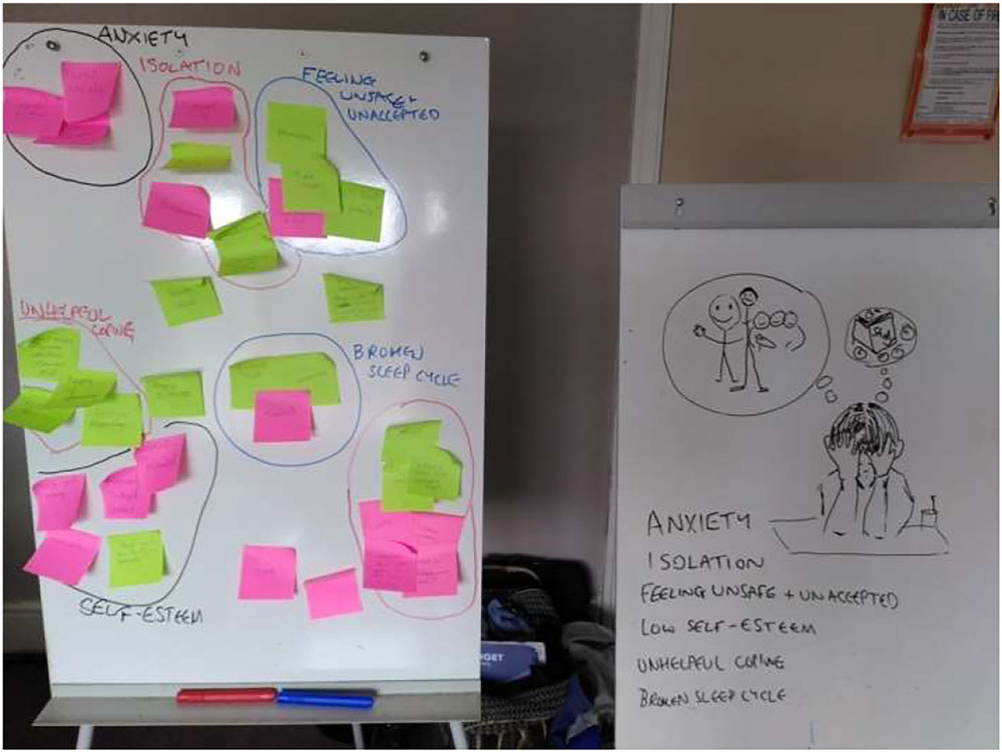
The persona emotional mapping exercise.

Initial workshops focused on establishing rapport and co‐production principles. Participants explored their understanding of psychosis, its media portrayal, and how to discuss it with young people. Pre‐prepared questions initiated discussions on the evolving meaning of psychosis and perceptions of youth mental health. The second workshop continued these themes and explored effective media like cartoons and interviews. A key activity was a facilitator‐led ‘persona‐emotional mapping’ exercise (Figure 3), adapted from business user experience design [64, 65], which enabled participants to discuss potential experiences of a young person developing psychotic symptoms without requiring personal disclosure [40].

#### 2.1.2 Workshops 3–4: Material Review and Core Components

**Figure 4:**
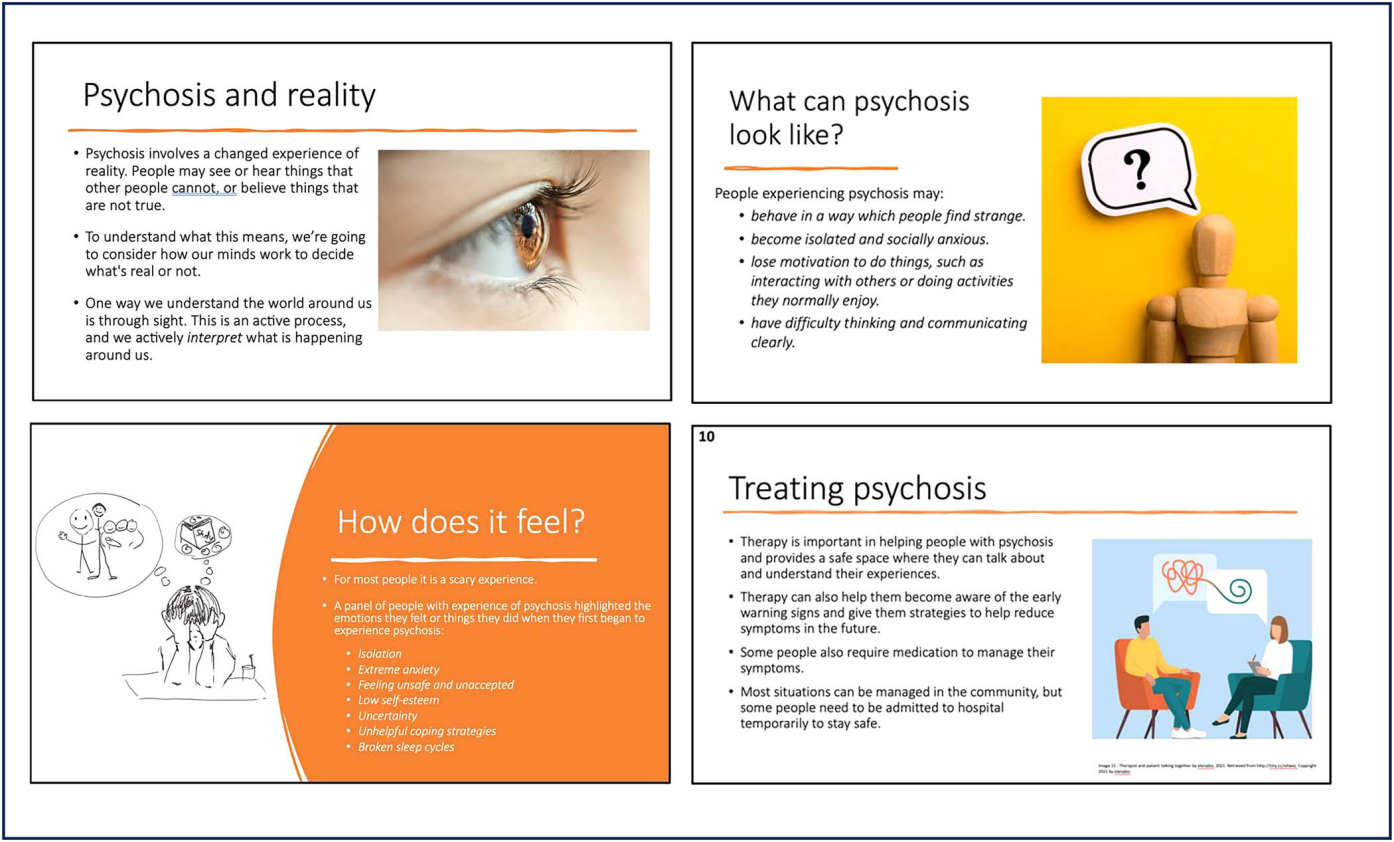
Example slides.

Workshop 3 involved reviewing existing youth‐focused materials on psychosis. The group acknowledged the impossibility of a completely neutral stance, emphasising a biopsychosocial rather than solely biomedical explanation. Consensus emerged on portraying psychosis as a spectrum connected to typical experiences to reduce stigma. Participants stressed the need for simple, age‐appropriate and nuanced language, avoiding generalisations and highlighting the diversity of experiences. Understandable and relatable examples were deemed crucial. Between workshops, facilitators synthesised workshop content and prior research into a module outline. Workshop 4 involved reviewing the draft, finalising core components and their sequence. A ‘Psychosis and Perception’ section was added as an introduction. Due to resource constraints, the group prioritised developing conventional educational material (presentation with teaching notes—Figure 4) for initial delivery, with potential for future expansion.

#### 2.1.3 Workshops 5 and 6—Reviewing and Refining Material

Later workshops concentrated on reviewing module drafts. Feedback was generally positive, with suggestions for increased interactivity, reordering for better flow, and framing psychosis as a misperception of reality.

### 2.2 Feedback From Clinicians and Teacher

Feedback from national and local Early Intervention in Psychosis (EIP) groups was positive, deeming the module age‐appropriate and high quality, with potential for use beyond schools (e.g., training of psychologists). Clinicians particularly valued the normalising approach and the dream analogy. Suggestions for improvement included: more information on drugs and psychosis; slide re‐labelling; language simplification; and inclusion of diverse real‐world examples, explicitly mentioning schizophrenia due to its higher recognition among young people. A consensus emerged that delivering the module over two sessions would be ideal for sufficient depth. Feedback from a teacher echoed the module's relevance and usefulness, with positive responses to the resources and facilitator‐led training support. While initially targeting 13–14 year‐olds, feedback indicated its suitability for older adolescents as well. The module structure and content are detailed in Supporting Information S1.

### 2.3 Experiences of PPIE

Before detailing reflections on the PPIE process, Table 1 sets GRIPP checklist items and reports how we addressed these.

**Table 1:** Overview of GRIPP2 item (SF) and how the study addressed them.

GRIPP items	What we did
Aim of PPIE	Co‐design of an educational module about psychosis for young people
Methods	– Co‐design process guided by the Double Diamond framework, involving six iterative workshops with experts‐by‐experience (EIP service users). – Workshops followed structured stages with flexible agendas and participant‐led input. – Consultations with clinicians, a national steering group and a teacher ensured the module's relevance; feedback was incorporated through iterative revisions. – Reflective journal kept by the primary researcher throughout the process.
Outcomes	– School‐based psychosis education module with teaching notes and interactive content.
How PPIE influenced the study/critical reflections	– Shaping module content and framing: Experts by experience guided key decisions on language, framing and topics, ensuring relevance and sensitivity to lived experience and suitability for young people. – Supported collaborative development: Co‐design principles were embedded throughout, with participants involved in agenda setting, activity design and iterative feedback.
Critical reflections	– Power dynamics and capacity challenges: Participants' limited experience in teaching and content creation, alongside fluctuating attendance, meant facilitators took on more responsibility, sometimes shifting the process from co‐design towards consultation. – Balancing idealism with pragmatism: The need to deliver a completed module within time constraints led to compromises in participation depth and scope, illustrating tensions common in co‐production efforts.

### 2.4 Balancing Facilitation and Participant Control

Engagement and rapport throughout the workshops were good, with participants motivated to contribute to the sessions. However, there were challenges in getting participants to drive the co‐design process. Firstly, there were challenges with recruitment and continuous participation due to ill‐health and other commitments. Secondly, while participants brought valuable lived experiences of psychosis, integral for the co‐design process, they lacked expertise in other areas. For example, they were less familiar with the clinical aspects of psychosis, how to create educational materials and how to facilitate a co‐design workshop effectively. Participants sometimes found it difficult to provide detailed input for specific aspects of the module development. Additionally, during the initial workshops, we began to realise that the amount of work needed to create the module required more support from facilitators than first anticipated. Due to these factors, the facilitators began to take on a more active role.

In order for participants to feel supported during this process, the facilitators ensured there was a continual dialogue with participants about how much control they felt comfortable taking on. An effort was made to share decision‐making, and participants were consulted at every appropriate juncture and given opportunities to guide the process. This included: agenda setting at the beginning of workshops, reviewing progress at the end of the workshop, and being involved in choosing activities. Feedback from participants indicated that they felt supported to take control of the process and their involvement as much as they were comfortable with and were happy with the majority of suggestions of ways to advance the workshops. Additionally, during conversations and activities, we felt ourselves being drawn into contributing our opinions and experiences from the perspective of clinicians and academics. In the spirit of co‐design, this seemed appropriate and helped create a rapport within the group and a safe space to discuss difficult subjects.

However, it was difficult to create this module collectively and effectively, without somewhat compromising our co‐design process. Overcoming unequal power dynamics between facilitators and participants was a constant challenge. This was exemplified when it came to synthesising materials. While participants were happy taking part in activities and discussion, they felt they did not have the skills to be involved in the module synthesis. This was problematic as although participants co‐created the content, both the creation of activities, which formed the basis of the content, and the synthesis of the module were done by researchers. Although this was necessary to continue the co‐design process and have a complete module by the end of it, it did take away ownership of the module from the participants, and in our eyes, the workshops became less co‐design and more consultative as the process continued. When we presented our draft materials in the latter workshops, there was the possibility that participants may have been unwilling or lacking the confidence to challenge us.

### 2.5 Managing Time and Sustaining Momentum

Midway through the workshops, we became keenly aware of our limited timescale and that it was necessary to start moving away from discussions and to think about how we wanted to create the material. A goal was proposed by facilitators that by the end of Workshop 4, the group would decide on what major topics they wanted to cover, and a rough idea of the content and messaging of each.

Careful thought is needed when creating a workshop with multiple sessions, about how best to space (or reduce the number of) sessions to balance maintaining momentum with participant well‐being. The group was finding the pace of the workshops challenging, having had a workshop for four consecutive weeks, leaving insufficient time for reflection and making it difficult to fit them into their schedules. Additionally, at this stage, one of the participant's mental health deteriorated, and they had to temporarily step aside from the workshops. Withdrawal by a second participant was also seen after Workshop 4. In both cases, their relevant mental health team was contacted, and support was put in place according to existing procedures. While it is uncertain whether these fluctuations were related to the workshops, monitoring participants' well‐being throughout a co‐design process is essential, especially when they draw on their own experiences, which may still be emotionally charged. The last two workshops were delivered over a longer period.

### 2.6 Integrating New Perspectives

As the process continued, a consensus was reached that it would be desirable to include more experts by experience in the co‐design process to increase the diversity of perspectives. A second round of recruitment was carried out between Workshops 4 and 5, and three new participants were included in the final three workshops. Integrating new participants into the co‐design process needed to be carefully managed. Firstly, unlike the original participants, who had time to get to know each other, establish rules of conduct and understand the co‐design process, the new participants were immediately placed in a consultative role to review the module. In hindsight, adjustment time should have been adequately prepared for in the workshop planning. Consequently, they may have felt less comfortable constructively criticising the material presented. Secondly, participants joining partway through the process sometimes shared thoughts or ideas that had been discussed in previous sessions. We needed to provide space for new participants to share their experiences and opinions whilst balancing this against the timely completion of a full module draft. Old and new participants worked well together and reviewed the module.

## 3 Discussion

### 3.1 Factor for Study Success

Based on our reflections and the outcomes of the co‐design process, the combination of expert by experience, academics and clinician involvement in the workshops, as well as consultation with clinician groups and teachers, was effective in creating this module. We believe several factors contributed to the success of this process. Firstly, adequate time and resources were given to plan the delivery of this project, from initial planning to finalisation of the module. This process took around a year and a half, which allowed time for sufficient planning and troubleshooting to take place. Generally, co‐approaches are quite complex and challenging procedures to implement successfully [66, 67], and allowing time and resources for planning has been shown to be beneficial [68]. The availability of sufficient time was crucial in fostering relationships with all relevant stakeholders, ensuring that everyone was willing and prepared to contribute effectively at the appropriate stages of the process. Secondly, clear networking and communication, leading to stakeholder buy‐in, was also key, as by bringing different stakeholder groups together with different expertise and perspectives, we were able to create a pool of experience and ideas that we could draw on. For example, the Local EIP group was fundamental in this process: having pre‐existing relationships with experts by experience, we could recruit for this co‐design process, help translate the finished material into Welsh, and serve as an invaluable feedback forum. The national EIP group and teacher also provided invaluable feedback about module content and its appropriateness for young people before module piloting.

### 3.2 Challenges

However, there were a number of interconnected challenges faced during the co‐design process. Firstly, the recruitment and retention of participants for the workshops were difficult throughout. Three participants dropped out of the initial workshop the day before commencement due to other commitments or ill‐health, and some participants attended the workshop sporadically. Flexibility was offered to the participants in terms of dates, but more choice in terms of time, such as evening or weekend, may have improved participation. Poor recruitment and small group sizes seem to be a challenge for many co‐approach mental health studies [69]. Smaller numbers of participants can be problematic as they may not represent the full range of experiences of what is being explored in the research. Additionally, although participants were renumerated for participating and travel, some potential participants may have felt that the pay provided did not meet their expectations for the amount of work involved.

Secondly, the desired outputs and time asked for from participants were quite ambitious for a co‐design project. Many co‐design processes with experts by experience focus on more limited goals such as: tailoring and testing existing digital designs; contributing to a component a larger project; or the process itself being a vehicle for empowering and improve outcomes for people with psychosis [69]. Where people with experience of psychosis were responsible for creating an intervention [41, 70, 71], the number of participants and other stakeholders participating in the workshop was usually higher. Due to the time and resource constraints, it was not possible to train participants before the co‐design process, as is recommended [68, 72]. If there is no time for training, it may be worth considering different ways of involvement. People have to have the skills and capacity to contribute. This is particularly important if, like in the case of our study, issues that participants may find distressing or emotionally taxing are explored. One possible solution to reduce the demands placed on experts by experience would be to include other stakeholders in the co‐design process. During the recruitment process, family members of people with lived experience of psychosis also expressed interest in participation. Although we decided against it at the time, it may be useful to explore different group compositions in future workshops. The inclusion of carers and family members can be seen in a study by Sin et al. [41] with people with experience of psychosis, and may add value to the co‐design process. Alternatively, family members and carers could be included in a consultation process in‐between some of the workshops [41, 70, 73].

Thirdly, throughout the process, there was a challenge of balancing the need to capture the co‐production process and doing things ‘by‐the‐book’, versus the practicality of ‘getting things done’ to achieve particular outcomes by the end of the workshops. This is something that has been seen in other co‐design processes [74, 75]. Due to the reality of having to produce a module at the end of the workshops, several pragmatic compromises were required. These resulted in a smaller number of participants and more involvement by facilitators in directing the workshops and synthesising the module. While a similar agile workflow has been shown to be successful in another project using a co‐approach with people with lived‐experience of psychosis [41], and efforts were made to share decision‐making in the current study, there was at times an unequal power dynamic which somewhat compromised the desired ‘co’‐approach. Unfortunately, power differentials are a common problem in other psychosis expert‐by‐experience co‐designed/produced research [60].

To our knowledge, this study marks an early attempt to utilise the GRIPP‐2 reporting checklist to systematically document the co‐design of mental health educational/anti‐stigma material for young people. While we did initially consider the GRIPP‐2 long form to ensure comprehensive reporting and enhanced transparency, we found that several sections of the long form were not applicable to our co‐design process. Furthermore, the level of detail required by the long form sometimes necessitated considerable interpretation and seemed difficult to align with our iterative workshop activities. Reporting on both the outcome of our study and the process of PPIE involvement can be challenging. However, the GRIPP‐2 is a robust tool with the potential to thoroughly document PPIE processes. An application can be intricate, requiring careful consideration to ensure it accurately reflects the dynamic nature of co‐design.

## 4 Conclusion

This study reports on the co‐design process of a psychosis educational intervention for young people, along with critical reflections on the process. Documenting this process provides insights into the practicalities of workshop facilitation, as well as some of the experiences and challenges researchers may face, and advice for any researcher thinking of using a co‐approach. Following this study, we are currently undertaking a pilot evaluation of this module to assess feasibility and effectiveness.

## Author Contributions

**William John Parrott:** conceptualisation, methodology, project administration, resources, writing – original draft. **Mike Jackson:** conceptualisation, methodology, supervision, writing – review and editing. **Anne Krayer:** conceptualisation, methodology, supervision, writing – review and editing.

## Conflicts of Interest

The authors declare no conflicts of interest.

## Supporting information

Supporting material Co‐design.

## Data Availability

The data that support the findings of this study are available from the corresponding author upon reasonable request.
