# Supplementary material for: Working With People With Experience of Psychosis to Co‐Design an Educational and Anti‐Stigma Psychosis Intervention for Schools
Source: Health Expect. 2025 Oct 17;28(5):e70333. doi: 10.1111/hex.70333 (PMC12532487; doi:10.1111/hex.70333)
Supplement: Supplementary file 1 — Supporting material Co‐design. [file HEX-28-e70333-s001.docx]

**Supplementary Material: Module structure and content**

**Part 1 – Understanding psychosis and its causes**

- **Reality and psychosis –** *To normalize psychosis as a changed perception of reality, we introduce the topic by talking about visual illusions, demonstrating how our experiences of the everyday can be ambiguous, distorted, or fictional.*
- **How we experience the world -** *We then illustrate through various examples how other factors such as mood, our other senses, and our beliefs and knowledge, can influence our interpretation of the world around us.*
- **The psychosis-stress relationship –** *We then contextualise psychosis within the stress and vulnerability bucket model (Brabban & Turkington, 2002), as well as it being a manifestation of altered perception.*
- **Prevalence and onset –** *Information on age of onset provided, as well as prevalence of both psychotic experiences (usually temporary with no lasting impact), and more prolonged psychotic illness.*
- **What is psychosis?** *Symptoms are then explained in simple, non-clinical language, along with some initial signs of how psychosis may present.*
- **Signposting and further information -** *information presented regarding further information and helplines available. Advice also presented to seek help from medical professional or trusted adult.*

**Part 2: Experiences of psychosis**

- **Further causes of psychosis –** *exploration of* *other possible causes of psychosis, such as genetics and differences in brain structure, drug use, and trauma.*
- **Experiences of psychosis** *– two fictionalized accounts of young people experiencing psychosis, highlighting perspectives from the individuals and their families.*
- **Famous people with psychosis -** *experiences of three celebrities, including videos of their discussions on psychosis.*
- **Treating psychosis***– explanation of therapies, emphasizing recovery and meaningful living even with ongoing symptoms.*
- **Acknowledge and disconfirming stereotypes -** *A true/false activity to challenge misconceptions about psychosis, followed by corrective information.*
- **Signposting and further information** *– repeated, as in previous module.*
